# Liver transplantation in ornithine transcarbamylase deficiency: A retrospective multicentre cohort study

**DOI:** 10.1016/j.ymgmr.2023.101020

**Published:** 2023-11-05

**Authors:** Berna Seker Yilmaz, Julien Baruteau, Anupam Chakrapani, Michael Champion, Efstathia Chronopoulou, Lee C. Claridge, Anne Daly, Catherine Davies, James Davison, Anil Dhawan, Stephanie Grunewald, Girish L. Gupte, Nigel Heaton, Hugh Lemonde, Pat McKiernan, Philippa Mills, Andrew A.M. Morris, Helen Mundy, Germaine Pierre, Sanjay Rajwal, Siyamini Sivananthan, Srividya Sreekantam, Karolina M. Stepien, Roshni Vara, Mildrid Yeo, Paul Gissen

**Affiliations:** a Genetics and Genomic Medicine Department, Great Ormond Street Institute of Child Health, University College London, London WC1N 1EH, UK; b Department of Paediatric Metabolic Medicine, Great Ormond Street Hospital for Children NHS Foundation Trust, London WC1N 3JH, UK; c Department of Inherited Metabolic Disease, Evelina Children's Hospital, Guy's and St Thomas' NHS Foundation Trust, SE1 7EH London, UK; d Department of Inherited Metabolic Disease, Division of Women's and Children's Services, University Hospitals Bristol NHS Foundation Trust, Bristol BS1 3NU, UK; e Leeds Teaching Hospitals NHS Trust, LS9 7TF Leeds, UK; f Birmingham Women's and Children's Hospital NHS Foundation Trust, B4 6NH, Birmingham, UK; g Paediatric Liver Gastroenterology and Nutrition Centre and Mowat Labs, King's College Hospital NHS Foundation Trust, WC2R 2LS, London, UK; h Institute of Liver Studies, Kings College Hospital, Denmark Hill, WC2R 2LS London, UK; i Willink Unit, Genetic Medicine, Manchester Academic Health Sciences Centre, Central Manchester University Hospitals NHS Foundation Trust, Oxford Road, Manchester M13 9WL, UK; j Adult Inherited Metabolic Diseases, Salford Royal NHS Foundation Trust, M6 8HD Salford, UK

**Keywords:** Liver transplantation, Ornithine transcarbamylase deficiency, Metabolic correction, Neurological outcome, Growth

## Abstract

Ornithine transcarbamylase deficiency (OTCD) is an X-linked defect of ureagenesis and the most common urea cycle disorder. Patients present with hyperammonemia causing neurological symptoms, which can lead to coma and death. Liver transplantation (LT) is the only curative therapy, but has several limitations including organ shortage, significant morbidity and requirement of lifelong immunosuppression. This study aims to identify the characteristics and outcomes of patients who underwent LT for OTCD.

We conducted a retrospective study for OTCD patients from 5 UK centres receiving LT in 3 transplantation centres between 2010 and 2022. Patients' demographics, family history, initial presentation, age at LT, graft type and pre- and post-LT clinical, metabolic, and neurocognitive profile were collected from medical records.

A total of 20 OTCD patients (11 males, 9 females) were enrolled in this study. 6/20 had neonatal and 14/20 late-onset presentation. 2/20 patients had positive family history for OTCD and one of them was diagnosed antenatally and received prospective treatment. All patients were managed with standard of care based on protein-restricted diet, ammonia scavengers and supplementation with arginine and/or citrulline before LT. 15/20 patients had neurodevelopmental problems before LT. The indication for LT was presence (or family history) of recurrent metabolic decompensations occurring despite standard medical therapy leading to neurodisability and quality of life impairment. Median age at LT was 10.5 months (6–24) and 66 months (35–156) in neonatal and late onset patients, respectively. 15/20 patients had deceased donor LT (DDLT) and 5/20 had living related donor LT (LDLT). Overall survival was 95% with one patient dying 6 h after LT. 13/20 had complications after LT and 2/20 patients required re-transplantation. All patients discontinued dietary restriction and ammonia scavengers after LT and remained metabolically stable. Patients who had neurodevelopmental problems before LT persisted to have difficulties after LT. 1/5 patients who was reported to have normal neurodevelopment before LT developed behavioural problems after LT, while the remaining 4 maintained their abilities without any reported issues.

LT was found to be effective in correcting the metabolic defect, eliminates the risk of hyperammonemia and prolongs patients' survival.

## Highlights

• Liver transplantation (LT) is an effective option for metabolic correction of OTC deficiency.
• All patients stopped protein restriction and ammonia scavengers after LT.
• Patients with neurodevelopmental problems before LT had persisting difficulties after LT.
• The clinical decisions on LT will need to take into consideration the potential benefits versus risks of morbidity, mortality and immunosuppression.

## 1 Introduction

Ornithine transcarbamylase (OTC) deficiency (OTCD) [MIM: 311250] is an X-linked defect of ureagenesis and the most common urea cycle disorder (UCD). [1] Recent studies based on newborn screening programmes and disease registries identified an incidence of between 1 in 56,500 and 1 in 77,000. [[2], [3], [4], [5], [6]] Ornithine transcarbamylase (OTC) (EC 2.1.3.3), encoded by the *OTC* gene, is essential for converting neurotoxic ammonia into urea in the liver and OTCD is characterized by ammonia accumulation. [7] Hyperammonemia can provoke irreversible damage to the central nervous system, leading to mental status changes, seizures, cerebral oedema and, in severe cases, death. [8]

OTCD has a wide phenotypic heterogeneity. Hemizygous males can present with either a neonatal onset (onset age ≤ 30 days) phenotype that correlates with the complete absence of enzyme activity or a late onset (onset age > 30 days) phenotype with partial enzyme activity. [9] Due to the random pattern of X-chromosome inactivation in hepatocytes, approximately 20% of heterozygous females are symptomatic with highly variable age of onset and clinical features. [10] Acute hyperammonemic decompensations can be precipitated by stressors and become a life-threatening event at any age and any stage of the disease. [9,11]

The standard of care consists of dietary protein restriction, ammonia scavengers, and arginine/citrulline supplementation. [12] However, this treatment does not prevent acute hyperammonemia episodes provoked by catabolic stress, such as intercurrent illness or fasting with continuing risk of severe neurological damage or death. [13] Liver transplantation (LT) is the only curative treatment due to its ability to correct the metabolic defect. Orthotopic LT is the standard recommended procedure. [14] However, it is a complex surgical procedure available only available in a few centres which has its own mortality and morbidity, limited donor availability and requires life-long immunosuppression. [14,15] Human heterologous liver cell transplant (LCT) has been proposed as an alternative or “bridge” therapy until LT, particularly in infants with highest risks and complications. However, LCT has shown only a modest and transient increase in ureagenesis and does not prevent all hyperammonemic episodes. [16]

LT provides improved metabolic stability, allows diet liberalization and discontinuation of ammonia scavengers and a better quality of life. [15,17] Nevertheless, LT does not reverse pre-existing neurological damage, hence, patients transplanted within the first 12 months of life might benefit more than children who were transplanted later in life. [[18], [19], [20], [21]] LT is recommended for patients with substantial OTC enzyme deficiency without severe neurological damage who do not respond to standard treatment and have a poor quality of life. The procedure should be performed while the patient is in a stable metabolic condition. [13] Therefore, the final decision of whether and when to perform LT depends on individual medical circumstances.

Due to the limited number of patients who have undergone LT, the reported literature lacks information about the current status of LT in OTCD. [22] We performed a retrospective, multicentre study to provide an up to date overview of transplanted OTCD patients in the UK in order to better understand patients' characteristics and global outcome.

## 2 Materials and methods

### 2.1 Patients

This multicentre retrospective study included 20 OTCD patients from 5 UK centres (Birmingham Women's and Children's Hospital, Bristol Royal Hospital for Children, Evelina London Children's Hospital, Great Ormond Street Hospital, and Salford Royal Hospital, Manchester) who underwent LT in three transplantation centres (Birmingham Women's and Children's Hospital, King's College Hospital and Leeds Teaching Hospitals) between 2010 and 2022.

Neonatal onset OTCD is characterized by episodic, life-threatening hyperammonemia within the first 28 days of life. The diagnosis is established by blood tandem mass spectrometry (MS/MS) and urine gas chromatography mass spectrometry (GC/MS) with biochemical findings including elevated glutamine, very low/absent citrulline and elevated urinary orotic acid. Although molecular analysis is the method of diagnostic evaluation for OTCD, it is not available for all patients.

Data were collected from the medical records of patients on standard clinical care and a standardized questionnaire was completed including information on gender, age, family history, method of diagnosis, initial presentation, number of hospital admissions, including intensive care admissions, medical and dietary management pre-transplant, age at LT, type of liver graft, clinical outcomes, diet and metabolic management post-transplant. Weight, and height were measured according to accepted standards and documented on sex-specific United Kingdom Growth Charts. Weight and height data before LT were the ones within one month before the procedure. Follow-up growth data was not available for all patients at all time points. No specific exclusion criteria were applied.

Pre- and post- LT neuropsychological status were assessed and recorded by paediatricians who had diagnosed and/or treated the patients. Developmental assessments and specialised tests were not available.

### 2.2 Ethical statement

Informed consent was obtained from legal guardians for participants registered at Great Ormond Street Hospital NHS Trust, Evelina London Children's Hospital and Salford Royal Hospital, Manchester (London-Bloomsbury National Research Ethics Committee: REC reference: 13/LO/0168, IRAS project ID: 95005, Study of Inherited Metabolic Disease). Bristol Royal Hospital and Birmingham Women's and Children's Hospital Research and Development Offices did not require participants to be consented due to collection of anonymous data.

### 2.3 Statistics

Data were described in their totality and according to recurrence status using median with interquartile range (IQR) or mean with standard deviation (SD) for continuous variables and number (percentage) for categorical variables. Patient survival was calculated from the date of LT to that of death or the final clinical visit. Graft survival was calculated from the date of LT to that of re-transplantation, death, or last visit if no re-transplantation. Cumulative survival rates at the 95% confidence interval were estimated with Kaplan–Meier survival analysis.

## 3 Results

### 3.1 Preoperative clinical characteristics

A total of 20 OTCD patients (11 males and 9 females) from 5 metabolic centres were enrolled in this study. Patients underwent LT in 3 transplantation centres between 2010 and 2022. Six patients (30%) had a neonatal presentation and all of them were males. 14 patients (70%) were diagnosed with a late onset presentation, including 9 females and 5 males.

All neonatal onset patients presented with encephalopathy and seizures in the first week of life and required hemofiltration. The mean peak ammonia levels at diagnosis were 1638 ± 610 μmol/L (N: 21–95 μmol/L) [23] and the median peak ammonia level was 1577 μmol/L (1000‐2808). There was no diagnostic delay, as all patients were diagnosed soon after the initial presentation (Table 1).

**Table 1:** Characteristics of OTCD patients underwent LT.

Patient No	Sex	Phenotype	Mutation	Age of initial presentation	Symptoms of initial presentation	Age of diagnosis
1	F	Late onset	c.422G > A (p.Arg141Gln)	12 months	Vomiting, poor feeding (12 m), altered consciousness, encephalopathy (15 m)	15 months
2	M	Late onset	c.931G > A (p.Val311Met)	12 months	Vomiting, lethargy	12 months
3	F	Late onset	c.78-1G > A	24 months	Vomiting, lethargy, liver dysfunction	24 months
4	F	Late onset	N/A	10 months	Vomiting (10 m), Encephalopathy (13 m)	13 months
5	F	Late onset	c.958C > T (p.Arg320Ter)	15 months	Vomiting, ataxia, tremor (15 m), lethargy, liver dysfunction (20 m)	20 months
6	M	Late onset	c.829C > T (p.Arg277Trp)	12 months	Developmental delay (12 m), Encephalopathy (20 m)	20 months
7	F	Late onset	Deletion of exons 1–10	17 months	Vomiting (17 m), lethargy, confusion (20 m)	20 months
8	M	Late onset	c.523G > A (p.Asp175Asn)	13 months	Seizures	13 months
9	F	Late onset	N/A	10 months	Vomiting, developmental delay (10 m), encephalopathy (12 m)	12 months
10	M	Late onset	c.918 A > C (p.Arg306Ser)	6 months	Vomiting (6 m), liver failure (8 m)	8 months
11	F	Late onset	c.422G > A (p.Arg141Gln)	24 months	Protein aversion, unsteady gait, and developmental delay (24 m), liver dysfunction, encephalopathy (36 m)	36 months
12	M	Late onset	c.829C > T (p.Arg277Trp)	No symptoms	No symptoms	Antenatal
13	F	Late onset	c.387-3C > G	36 months	Mild learning difficulties and behavioural problems (36 m), encephalopathy (72 m)	72 months
14	F	Late onset	Deletion of exons 1–10	24 months	Vomiting and behavioural problems (24 m), lethargy, confusion (30 m)	30 months
15	M	Neonatal	c.422G > A (p.Arg141Gln)	5 days	Encephalopathy, seizures	6 days
16	M	Neonatal	N/A	3 days	Encephalopathy, seizures	5 days
17	M	Neonatal	c.829C > T (p.Arg277Trp)	4 days	Encephalopathy, seizures	7 days
18	M	Neonatal	c.482 A > G (p.Asn161Ser)	3 days	Encephalopathy, seizures	3 days
19	M	Neonatal	N/A	3 days	Encephalopathy, seizures	3 days
20	M	Neonatal	Deletion of exons 5–9	3 days	Encephalopathy, seizures	5 days

F: Female; M: Male; N/A: Not available; m:month.

Presenting symptoms were variable for late onset patients. 12/14 patients (86%) presented with neurological symptoms including alterations in consciousness, seizures, developmental delay, and ataxia. Vomiting was also one of the most prominent symptoms reported in 9 out of 14 patients (64%). Vomiting and/or developmental delay were reported prior to neurological symptoms in 10/14 patients (71%), but as these symptoms are non-specific, they did not lead to diagnosis. Whilst only one patient presented with acute liver failure (characterized by severe acute hepatic dysfunction with an International Normalized Ratio (INR) ≥ 1.5 and encephalopathy in the absence of chronic liver disease), 3/14 patients had liver dysfunction during the initial episode. Median age of onset was 13 months (range 6–36 months) and median age at diagnosis was 20 months (range 8–72 months) after the exclusion of one asymptomatic patient who was antenatally diagnosed and treated prospectively immediately after birth due to an index case in the family (Table 1). The ammonia level at diagnosis was known for 10/14 patients and the mean peak value was 201 ± 90 μmol/L (N for children over 1 month of age < 55 μmol/L). [24] The median peak ammonia level was 220 μmol/L (range 47–339 μmol/L).

Genetic analysis of OTC was available for 16 patients out of 20 (Table 1). The most common mutations reported in our cohort were c.829C > T (p.Arg277Trp) and c.422G > A (p.Arg141Gln), both reported in 3/16 (19%) patients.

All patients were on protein-restricted diet before LT (Table 2). The median natural and total protein intake were 1.12 g/kg/d (range 0.5–1.5 g/kg/d) and 1.17 g/kg/d (range 0.6–1.8 g/kg/d) for neonatal onset and late onset patients, respectively. (Table 2) 60% (12/20) of patients required nasogastric or gastrostomy tube feeding before having LT. All patients were on ammonia scavengers before LT; 15 out of 20 patients were receiving dual scavenger therapy including a combination of sodium benzoate and sodium/glycerol phenylbutyrate, 4/20 were treated with only sodium benzoate and 1 patient was receiving phenylbutyrate only. All patients were treated with arginine and/or citrulline supplementation before LT.

**Table 2:** Metabolic parameters for OTC deficiency patients before and after liver transplantation.

Patient No	Ammonia at diagnosis (μmol/L)	Number of hospital admissions from diagnosis to LT	Number of hospital admissions with hyperammonemia (≥100uM) from diagnosis to LT	Peak ammonia levels during the follow up (μmol/L)	Pre-LT natural/total protein intake (g/kg/day)	Age of LT (m)	LT Centre	Procedure	Follow up period	Post-LT protein intake
1	176	Multiple	Multiple	150	0.85/1	156	LTH	LDLT	11y	NR
2	N/A	10	3	165	0.6/0.6	152	KCLH	DDLT	7y	NR
3	264	3	1	105	1/1	35	KCLH	DDLT	5y, re-transplanted at 19d	NR
4	249	Multiple	10	230	1/1	77	BCH	DDLT	12y, re-transplanted at 79 m	NR
5	339	Multiple	3	212	1.5/1.5	56	BCH	DDLT	9y	NR
6	84	Multiple	0	72	1.5/1.5	41	BCH	DDLT	9y	NR
7	81	10	7	289	1/1	71	BCH	DDLT	6 h, deceased	NR
8	207	6	5	168	1.5/1.5	70	BCH	DDLT	1.5y	NR
9	249	Multiple	Multiple	200	1.5/1.5	79	BCH	DDLT	12y	NR
10	N/A	3	0	61	1.2/1.2	35	KCLH	DDLT	9y	NR
11	N/A	3	2	289	1.5/1.5	62	BCH	DDLT	13y	NR
12	47	1	0	<100	1.5/1.5	39	BCH	LDLT	3y	NR
13	300	Multiple	0	80–90	1.1/1.5	119	KCLH	DDLT	12y	NR
14	220	22	15	312	0.6/1	51	KCLH	DDLT	4y	NR
15	1354	10	3	140	0.8/1.1	10	KCLH	LDLT	2.5y	NR
16	1069	Multiple (Received 5 hepatocyte infusions before LT)	2	116	1.2/1.8	6	KCLH	DDLT	11y	NR
17	1800	4	0	68	1.45/1.58	7	KCLH	DDLT	4y	NR
18	1800	13	6	2800	0.5/0.9	11	KCLH	LDLT	3y	NR
19	1000	4	2	294	1/1	24	KCLH	DDLT	1y	NR
20	2808	2	0	92	1.14/1.14	14	KCLH	LDLT	1y	NR

LT: Liver transplantation; Multiple: ≥5; LTH: Leeds Teaching Hospitals; KCLH: Kings College Hospital; BCH: Birmingham Children's Hospital; LDLT: Living related donor liver transplantation; DDLT: Deceased donor liver transplantation; h: hours; d: days; m: Months; y: Years; NR: Not restricted.

All patients had >1 hospital admissions from diagnosis to LT except the patient who was antenatally diagnosed and received medical treatment prospectively after birth (Patient 12). 14/20 patients (70%) had 1 or more hospital admissions with ammonia levels ≥100 μmol/L despite being under standard of care management. One of the neonatal onset patients had hepatocyte transplantation (given in 5 separate infusions) and then had an orthotopic LT at the age of 6 months (Patient 16) (Table 2).

While all neonatal onset patients had moderate to severe neurodevelopmental delay before LT, only 10/14 of late onset patients (71%) had a varying degree of delay. Speech delay was the most prominent component in more than half of these patients. Learning difficulties and behavioural problems were recorded together in 15% of the patients (Table 3).

**Table 3:** Neurocognitive status and MRI findings before and after liver transplantation.

Patient No	1	2	3	4	5	6	7	8	9	10	11	12	13	14	15	16	17	18	19	20
NCS before LT	N	ADHD, Autism, significant behavioural difficulties	DD	N	N	Global DD, significant speech delay	Speech delay, motor delay	N	Behavioural problems	Cognitive dysfunction	Moderate learning difficulties	N	Mild learning difficulties	Mild DD (mainly speech)	Mild DD (mainly speech)	DD	Mild motor delay	Global DD	Significant DD, low muscle tone	DD, mainly speech
NCS after LT	ADHD	Behavioural problems, improved	DD but making progress	N	N	Global DD, not able to walk independently	Deceased	N	Needs counselling psychiatric support	At a special school, cognitive impairment, behavioural problems, learning difficulties	Moderate learning difficulties not improved	N	Mild difficulties attending normal school	Mild DD	Mild DD especially speech delay	DD, attends special school, wheelchair bound	No significant concerns, mild DD	Global DD, behavioural problems, seizures	Central tone has improved, more active, swallowing improved, still nonverbal	Walking unsupported, mild speech delay
Brain MRI before LT	Minor atrophic changes in occipital and frontal lobes	Generalised atrophy	N/A	N/A	N/A	High signal in the peritrigonal white matter that does not extend to the ventricular wall	N/A	Multilobar extensive increased signal intensity with restricted diffusion in the cortical grey matter and subcortical white matter.	N/A	N/A	N/A	N/A	N/A	N/A	N/A	N/A	Normal brain parenchyma. Ventricles are minimal prominent	N/A	N/A	N/A
Brain MRI after LT	Mild cerebral atrophy	N/A	N/A	N/A	N/A	Myelination delay, diffuse high T2 signal in central white matter of both hemispheres mainly in parietal and occipital. Ventricles are prominent, mild diffuse cerebral atrophy.	Deceased	N/A	N/A	N/A	N/A	N/A	N/A	N/A	N/A	N/A	N/A	N/A	N/A	N/A

MRI: Magnetic resonance imaging; NCS: Neurocognitive status; LT: Liver transplantation; ADHD: Attention deficit hyperactivity disorder; DD: Developmental delay; N:Normal; N/A: Not available; CSF: Cerebrospinal fluid.

Five patients had a brain MRI scan before LT. Only one of them was reported as normal. MRI findings are shown in Table 3.

### 3.2 Age at liver transplantation

4/6 neonatal onset patients (66%) were transplanted in the first year of life and the remaining 2/6 (34%) underwent LT in the second year of life with median age at LT being 10.5 months (range 6–24 months). Of those with late onset presentation, the median age of LT was 66 months (range 35–156 months) (Table 2). The median time from the initial presentation to LT was 54 months (range 11–144 months) in late onset females and 43 months (range 29–124 months) in late onset males. 1 patient who was antenatally diagnosed due to an index case in the family was treated prospectively and remained asymptomatic. (Patient 12).

The indication for LT was the presence (or family history) of recurrent metabolic decompensations leading to hyperammonemia despite standard medical therapy. The aim of LT was to prevent the onset or progression of neurological damage and improve the quality of life.

### 3.3 Graft type

25% (5/20) of patients (4 neonatal onset and 1 late onset) received grafts from living related donors whereas 75% of the cases (15/20) (2 neonatal onset and 13 late onset) had graft from cadaveric donors (Table 2). 1 patient (5%) had auxiliary LT. 17/20 patients (85%) had split liver grafts, 2/20 (10%) had whole liver grafts and graft type was not recorded for 1 patient (5%).

### 3.4 Surgical outcome and survival after liver transplantation

**Fig. 1:**
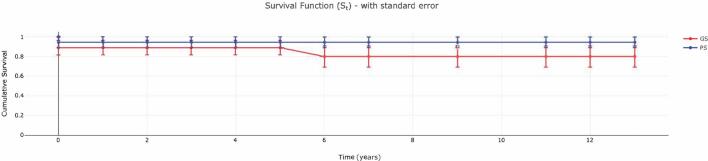
Cumulative posttransplant patient and graft survival rates in 20 patients with OTCD undergoing liver transplantation. Time is in years. GS: Graft survival; PS: Patient Survival.

The postoperative follow-up period ranged from 6 h to 13 years. Overall patient survival rate was 95% after LT (Fig. 1). Of the 20 OTCD patients who had LT, only one patient died, which occurred 6 h after LT due to hepatic artery thrombus and graft failure. 13/20 patients (65%) had at least one complication following LT. Acute rejection (4/20), hepatic artery thrombus (3/20), biliary leak (3/20) were the most frequent complications. 2/20 patients developed renal dysfunction after LT due to the nephrotoxicity of immunosuppressive agents. 2 out of 20 patients reported to have pleural effusion as a post-transplant complication.

Graft survival rate at 1 year was 90% (Fig. 1). 2/20 patients (10%) required re-transplantation. One of these patients had graft rejection after a severe cryptosporidium infection, graft survival was 79 months. She had a re-transplantation with a cadaveric split graft and after 68 months follow-up she is alive without any current problems (Patient 4). The other patient had a hepatic artery thrombus and rejection after the first LT and graft survival was 19 days. She was re-transplanted with a split cadaveric graft, and she had no further problems in the 58 months follow-up period (Patient 3).

### 3.5 Metabolic outcome after liver transplantation

All patients discontinued dietary restrictions and ammonia scavengers after LT. Only one patient has been receiving arginine and citrulline supplementation after LT. None of the patients had further metabolic decompensations after LT. 5/12 patients who required gastrostomy or nasogastric tube feeding before LT transitioned to exclusively oral feeding afterwards.

Growth parameters after LT are summarised in Table 4. Follow-up data was not available for all patients at all time points. 3 patients had a fall in weight centile by two major percentiles (percentile markers 95, 90, 75, 50, 25, 10, and 5) over 3 years' time post-LT but only patient's weight for age was below 5th centile. Height centile decreased by two major percentiles in 4 patients at 3rd year follow up post-LT and 4 patients' height centiles for age was under 5th centile 3 years after LT.

**Table 4:** Growth parameters before and after liver transplantation.

Patient No	1	2	3	4	5	6	7	8	9	10	11	12	13	14	15	16	17	18	19	20
Weight/height centile before LT	92/96	57/7	50/22	50/25	50/50	50/25	62/35	25/50	50/50	91/75	9/2	25 /25	91/75	71/8	<2/11	7/N/A	61/50	99/N/A	32/55	91/75
Weight and height centile 6 m after LT	N/A	32/5	N/A	50/25	50/50	50/25	Deceased	25/50	25/25	75/75	25/9	25/50	95/N/A	38/N/A	2/12	2/2	14/5	98/69	5/25	75/50
Weight and height centile 1y after LT	N/A	55/3	25/41	50/25	25/50	50/9	Deceased	25/50	25/25	75/75	25/9	25/50	N/A	12/N/A	N/A	N/A	7/2	55/46	11/25	N/A
Weight and height centile 2y after LT	N/A	70/3	25/18	50/25	25/50	50/9	Deceased	N/A	25/25	75/75	25/9	25/50	N/A	N/A	N/A	N/A	3/3	59/47	N/A	N/A
Weight and height centile 3y after LT	84/82	81/4	36/N/A	50/25	25/50	25/2	Deceased	N/A	25/25	75/75	25/9	25/50	N/A	9/8	7/4	30/N/A	3/2	68/N/A	N/A	N/A

m: month; y: year; N/A: Not available.

### 3.6 Neurodevelopmental outcome after liver transplantation

According to paediatricians' clinical decision without standard developmental tests, sixteen (15/20) patients who had neurodevelopmental delay before LT continued to have problems after LT. Four out of 5 late onset patients who had normal neurodevelopmental status before LT maintained their abilities after LT, neurodevelopmental deterioration was not observed. One other late onset patient who did not have any problems before LT, developed attention deficit hyperactivity disorder (ADHD) during the follow-up (Table 3).

Two patients had a follow-up brain MRI post-LT, both had no significant changes compared to the previous scans (Table 3).

## 4 Discussion

LT is the only available curative treatment for OTCD as it can restore the OTC enzyme in the liver and provide sufficient enzymatic activity for ureagenesis. [13,15] It is recommended for severe patients who are not well-controlled with medical treatment when they are in a stable metabolic state and ideally before neurological damage occurs. [20] Due to technical complexity, donor shortage, requirement for life-long immunosuppression, LT is not widely utilised for OTCD patients. [14,15] Therefore, there is a paucity of information in the literature on patient selection, time of transplantation, graft and donor type, surgical, clinical, neurological outcome, and long-term follow-up. To the best of our knowledge, this paper describes one of the largest cohorts of OTCD patients who underwent liver transplantation.

Age of onset and initial symptoms were very homogenous in the neonatal onset patients and there was no significant diagnostic delay reported. There is a wide variability of symptoms and age of onset in late onset patients as well as in the delay in diagnosis. This is in agreement with previous results from the Urea Cycle Disorders Consortium (UCDC) and European registry and network for the intoxication type Metabolic Diseases (*E*-IMD)registries. [25] Age at disease onset and peak ammonia levels of the initial presentation were found to be strongly associated with the neurological outcome. [26]

Mean peak ammonia levels at diagnosis were almost 7-fold higher in the neonatal onset group compared to the late onset ones. It was proven that initial mean peak ammonia levels are negatively correlated with long-term neurodevelopmental outcomes. [27] This is in line with our cohort, where all neonatal patients had a neurodevelopmental delay before LT.

Slightly more than half of the patients (55%) in our cohort were males, which is similar to UCDC and E-IMD cohorts. [25] However, in the patient cohorts from China (almost 80%) and Poland (87.5%) the majority of patients who underwent LT were females. [28,29] This may be due to the higher survival rates of female patients and national differences in management.

Optimal timing for LT in OTCD is very important for better outcome. In a previous study, it was recommended that patients who have mean peak ammonia concentrations ≥300 μM should be considered for LT as further neurological damage can be prevented. [30]. LT is recommended to be performed from 3 to 12 months of age when body weight exceeds 5 kg. [13] However, due to a particularly brittle nature of the neonatal onset and phenotypic heterogeneity of the late onset patients, the decisions on LT should be made on an individual basis after a multidisciplinary team discussion in order to achieve the best possible outcomes. In our study, although all neonatal onset patients presented within the first week of life, only 66% of them underwent LT in the first year of life and the remaining 34% had LT between the first and second year of life. As age of initial presentation varies in late-onset patients, there is also a heterogeneity in age of LT. Duration between the initial presentation to LT is also longer in late onset patients. [14,29] In our cohort, none of the late onset patients underwent LT in the first two years of life and the median time from the initial presentation to LT was 54 months in this group.

The accepted goal for LT in OTCD is to prevent the onset or progression of neurological damage. The Japanese Ministry of Health has established a scoring system for evaluation of the indication for LT in metabolic disorders. This grading system is based on the involvement of liver; the effectiveness of standard medical treatment; the quality of life; and the mental/physical status. [31] Although our patients were not graded on this scale, the decisions were based on the frequency of hyperammonemic decompensations despite medical treatment, neurological status and quality of life.

As patients achieve metabolic stability without further protein restriction or ammonia scavengers after LT, most patients and their care givers determine a consequent improvement in their quality of life. [32] Citrulline levels remain low post LT due to intestinal enzyme deficiency. [27] Our results suggest that it has no clinical significance as none of the patients had hyperammonaemia and linear growth was mostly normal. Only one patient received arginine and citrulline supplementation after LT as her plasma arginine concentration decreased several months after the LT and plasma citrulline concentration decreased many years after the procedure.

Despite good metabolic control, LT does not reverse pre-existing neurological impairment. [18,20,21,33] It was shown that early LT results in better neurological outcome. [34] LT had varying effects on different neuropsychological domains in late onset patients. While there was little change in overall IQ and behavioural problems tended to remain after LT, improvement has been reported in memory. [35] Early LT was shown to provide better neurological outcome with an average developmental quotient of 67 during a 45-month follow up period in 5 children diagnosed with UCDs. [20] In our study, all patients who had neurological impairment before LT had persisting neurological problems after LT. One of the late onset patients who did not have any neurodevelopmental problems prior to LT, developed ADHD after LT, which is most likely to be a complication of underlying OTCD. But, in our cohort, particularly in late onset patients, there was a gap between the initial presentation and LT. This may be the cause of insufficient neurological improvement. Moreover, patients who had relatively low ammonia levels both at the initial presentation and during the follow up may have a poor neurological outcome after LT (patient 6). This may be due to the delay in LT as the patients already have neurological impairment before LT.

One of the neonatal onset patients had hepatocyte transplantation before LT. Hepatocyte transplantation has been utilised to restore liver function and to act as a bridge until LT becomes a feasible option. It is a less invasive procedure compared to LT and can be performed at the bedside. Although safety of the procedure has been shown and short-term efficacy has been proven, it still requires immunosuppression and re-administration hence it is currently only used as a temporary measure. [36,37]

Orthotopic LT is the standard recommended procedure. However, auxiliary LT, where the right or left lobe of the patient liver is replaced with a healthy donor liver, has successfully been used to treat several metabolic liver diseases that do not cause liver fibrosis. [38] In our cohort, auxiliary transplantation was only used in one case, reflecting that this is a technically demanding procedure. [39] This approach could be considered as another temporary measure until such time when the option of gene therapy targeting the native liver becomes available. [40]

75% of patients had received LT from a deceased donor, while 25% had living related donors. Although deceased donor LT is common for OTCD, patient and graft survival were similar in both living related and deceased donor LT. [41] LDLT carries risks for the donors, but it can be a life-saving solution when there is a shortage of donor livers. [42] There have been successful reports of LDLT from asymptomatic heterozygous female donors, although there is a remaining concern that X-inactivation in the transplanted liver cells could provide insufficient OTC correction. [31,32,43,44] The above concern aside LDLT provides flexibility for the timing of LT and some complications such as hepatic artery thrombosis occur less frequently in LDLT compared to DDLT. [41]

OTCD is a severe disease especially in neonatal onset phenotype with 30% 5-year survival rate with standard of care. [45] Most survivors suffer from moderate to severe neurological impairment. [32,45] Although LT is a surgical procedure with its own risks, survival rates 5 years post-transplant have been reported as over 90% in different studies. [32,39] In our study, patient survival rate was 95% at 5 years after LT. Prior studies suggest that recipient age, graft type, and diagnosis affect graft survival. [46] Recent data from the United Network for Organ Sharing (UNOS) database reported a 90.4% 1-year graft survival and 85.2% 5-year graft survival for paediatric UCD patients who had received LT. [47] This is very similar to our results. While significant improvements have been achieved in mortality by advancing surgical techniques and medical management, complication rates remain relatively high after LT.

This is the first overview of current practice on LT in OTCD patients in the UK. Although this study has a large number of patients with a long-term follow-up, there are several limitations. The cohort is heterogeneous and there is no control group to compare to those that have received conventional treatment. Due to the retrospective nature of the study, some data were missing or unavailable. The method by which neurocognitive status was reported does not represent measures from a standardized and validated assessment tool, therefore, it is not quantitative and objective evaluation is difficult. Hence, these limitations warrant additional studies with prospective follow-up. More detailed neurocognitive follow-up and comparison with a non-transplanted cohort would improve our understanding of the neuroprotective potential of LT.

As LT is not widely available for OTCD patients and has its own risks, additional novel therapies are in development to treat OTCD. [48,49] Based on successful preclinical studies, AAV gene therapy and mRNA therapy are at the stage of clinical translation. [[50], [51], [52]] Proof-of-concept studies using gene editing or cell therapy strategies in OTCD are also promising. [53,54] Thus LT may potentially be replaced by emerging gene therapy interventions in the future.

## 5 Conclusion

In conclusion, LT is effective in metabolic correction of OTCD. It is essentially lifesaving as it prevents hyperammonemia. It also provides a significant improvement in the quality of life by eliminating the need for dietary protein restriction and ammonia scavengers. However, LT does not reverse the existing neurological damage. Therefore, timing of LT is critical since patients should ideally be transplanted before neurological damage occurs. Nevertheless, the early neonatal LT is technically challenging, hence there is a particularly high unmet need for novel therapy development. Further research is needed to establish clear guidelines and optimize the outcomes of LT in OTCD.

## Funding

This work was supported by the 10.13039/501100000265Medical Research Council grant, reference: MR/S019111/1. PG is supported by a National Institute of Health Research Senior Investigator Award (Reference NIHR202370). JB is supported by a Medical Research Council Clinician Scientist Fellowship MR/T008024/1. P.G. and P.M. are supported by the NIHR Great Ormond Street Hospital Biomedical Research Centre (the views expressed are those of the author(s) and not necessarily those of the NHS, the NIHR or the Department of Health).

## Author contributions

B.S.Y. and P.G. conceived the idea and designed the study. J.B., A.C., M.C., E.C., L.C.C., A.D., C.D., J.D., A.D., S.G., G.L.G., N.H., H.L., P.M.K., P.M., A.A.M.M., H.M., G.P., S.R., S.S. (Siyamini Sivananthan), S.S. (Srividya Sreekantam), K.M.S., R.V. and M.Y. have been involved in data collection. B.S.Y. analysed the data and wrote the manuscript, P.G. supervised the study. All authors have read and agreed to the published version of the manuscript.

## Declaration of Competing Interest

PG is an academic co-founder of Bloomsbury Genetic Therapies, UCL spinout developing a gene programme in OTC deficiency. JB receives research funding from Moderna Therapeutics, developing gene therapy for urea cycle defects.

## Data Availability

The data that has been used is confidential.
